# Viperin is anti-viral *in vitro* but is dispensable for restricting dengue virus replication or induction of innate and inflammatory responses *in vivo*

**DOI:** 10.1099/jgv.0.001669

**Published:** 2021-10-19

**Authors:** Wisam-Hamzah Al Shujairi, Luke P. Kris, Kylie van der Hoek, Evangeline Cowell, Gustavo Bracho-Granado, Tahlia Woodgate, Michael R. Beard, Jillian M. Carr

**Affiliations:** ^1^​ College of Medicine and Public Health, Flinders University, Adelaide, SA, Australia; ^2^​ Department of Clinical Laboratory Sciences, College of Pharmacy, University of Babylon, 51001 Hilla, Iraq; ^3^​ School of Biological Sciences, Research Centre for Infectious Diseases, The University of Adelaide, Adelaide, SA 5005, Australia

**Keywords:** dengue virus, interferon response, inflammatory response, neurovirulence, viperin

## Abstract

Viperin has antiviral function against many viruses, including dengue virus (DENV), when studied in cells in culture. Here, the antiviral actions of viperin were defined both *in vitro* and in a mouse *in vivo* model of DENV infection. Murine embryonic fibroblasts (MEFs) derived from mice lacking viperin (vip^−/−^) showed enhanced DENV infection, accompanied by increased IFN-β and induction of ISGs; IFIT1 and CXCL-10 but not IRF7, when compared to wild-type (WT) MEFs. In contrast, subcutaneous challenge of immunocompetent WT and vip^−/−^ mice with DENV did not result in enhanced infection. Intracranial infection with DENV resulted in body weight loss and neurological disease with a moderate increase in mortality in vip^−/−^ compared with WT mice, although this was not accompanied by altered brain morphology, immune cell infiltration or DENV RNA level in the brain. Similarly, DENV induction of IFN-β, IFIT1, CXCL-10, IRF7 and TNF-α was not significantly different in WT and vip^−/−^ mouse brain, although there was a modest but significant increase in DENV induction of IL-6 and IfI27la in the absence of viperin. NanoString nCounter analysis confirmed no significant difference in induction of a panel of inflammatory genes in WT compared to vip^−/−^ DENV-infected mouse brains. Further, polyI:C stimulation of bone marrow-derived macrophages (BMDMs) induced TNF-α, IFN-β, IL-6 and Nos-2, but responses were not different in BMDMs generated from WT or vip^−/−^ mice. Thus, while there is significant evidence of anti-DENV actions of viperin in some cell types *in vitro*, for DENV infection *in vivo* a lack of viperin does not affect systemic or brain susceptibility to DENV or induction of innate and inflammatory responses.

## Introduction

Central to the type I interferon (IFN) antiviral response is induction of interferon-stimulated genes (ISGs), such as viperin (virus inhibitory protein, endoplasmic reticulum-associated, interferon-inducible) [1–3]. Viperin expression is increased in response to a number of viral infections and has antiviral actions *in vitro*, including against human cytomegalovirus (HCMV) [4] chikungunya virus (CHIKV) [5], influenza A virus (IAV) [6], hepatitis C virus (HCV) [7] and several members of the family *Flavivirus*, such as West Nile virus (WNV) [8, 9], tick-borne encephalitis virus (TBEV) [9], dengue virus (DENV) [10] and zika virus (ZIKV) [8]. Specifically, DENV infection induces viperin mRNA and when viperin is ectopically expressed, DENV RNA levels are reduced [10], potentially by an interaction with the DENV NS3 protein and the viral replication complex [11]. While viperin is induced in the mouse brain following DENV-2 intracranial (i.c.) infection [12] and in the mouse eye following systemic infection in interferon (IFN)-deficient mice [13], the antiviral role of viperin against DENV infection *in vivo* in immunocompetent animals is undefined. For other viral infections, *in vivo* viral challenge in the absence of viperin has resulted in varied outcomes; namely, enhanced CHIKV infection and CHIKV-induced footpad swelling [5], enhanced WNV mortality and infection in the brain [14], lack of an effect on IAV infection in the lung [15] and partial antiviral effect in transiently reducing viral load and spread during Langat virus (LGTV) infection in the brain [16].

Here, the previously described *in vitro* anti-DENV role of viperin was confirmed using primary murine embryonic fibroblasts (MEFs) from viperin null mice. Viperin, however, was not required for efficient induction of IFN-β and other ISGs in the *in vitro* MEF system and not essential for restricting DENV infection *in vivo*, where lack of viperin did not enhance systemic or i.c. DENV replication or disease, or affect the inflammatory profile in the brain. Thus, while viperin is anti-viral against DENV *in vitro*, *in vivo* the actions of viperin are dispensable for antiviral activity and, at least in the brain, also the induction of inflammatory responses.

## Methods

### Cells, virus stocks and *in vitro* infection

Primary MEFs and bone marrow-derived macrophages (BMDMs) were generated from wild-type (WT) and vip^−/−^ embryos, as described previously [8]. Huh7 control and viperin shRNA are as described previously [7]. WT and vip^−/−^ MEFs were infected at a multiplicity of infection (m.o.i.) of 0.1 for 90 min with Mon601, a full-length DNA clone of the DENV-2 strain New Guinea C [17]. Huh7 control or viperin shRNA-expressing cells were similarly infected with D2Y98P-PP1, (GenBank accession #JF327392) originating from a 2005 Singapore clinical isolate (kindly provided by Associate Professor Sylvie Alonso from the National University of Singapore and by the National Environment Agency). Both DENV-2 strains were amplified in C6/36 insect cells and titred by plaque assay in Vero (African green monkey kidney) cells as described previously [12]. Cell culture-infected supernatants were collected and titrated by plaque assay on Vero cells and quantified as plaque-forming units (p.f.u. ml^−1^). Total RNA was extracted from cells and viral RNA and innate responses quantitated by qRT-PCR as below.

### *In vivo* DENV challenge

For systemic DENV challenge, 10^4^ p.f.u. of D2Y98P-PP1 was administered subcutaneously in 100 µl volume to 4–5-week-old WT and vip^−/−^ mice (*n*=5 each; DENV and mock). Mice were monitored daily for 6 days post-infection (p.i.) and then humanely killed with tissues (brain, liver and spleen) and blood being harvested. For i.c. DENV challenge, 3–4-week-old WT and vip^−/−^ mice were anaesthetized by isoflurane inhalation and infected by i.c. injection with 800 p.f.u. of DENV-2 Mon601 diluted in phosphate-buffered saline (PBS), in a volume of 10 µl (*n*=12 each). Mock-infected animals were similarly anaesthetized and injected i.c. with 10 µl PBS (*n*=8 each). Mice were monitored for weight loss and signs of neurovirulence, as described previously [12]. At the termination of the experiment or ethical end point, animals were killed by isoflurane inhalation and humane decapitation. Brain tissues were harvested; ipsilateral (relative to injection) hemispheres were resuspended in TRIzol reagent (Ambion Life Technologies) for RNA extraction and real-time quantitative PCR (qRT-PCR) analysis, and contralateral hemispheres were fixed for histological analysis as below.

### RNA extraction and RT-PCR

RNA was extracted from cells or tissue using TRIzol, DNase I treated (Zymo Research) and quantitated by spectrophotometry (NanoDrop lite, Thermo Scientific); 0.5 µg RNA was reverse-transcribed using random hexamers and subjected to real-time qRT-PCR. qRT-PCR was carried out using SYBR green with 2 µM of each primer, as previously described [12], with primers as shown in Table 1. Real-time qRT-PCR was performed using a Rotor-Gene real-time PCR system (Qiagen) as follows: 1 cycle of 95 °C for 5 min, 40 cycles of 95 °C for 15 s, 58 °C for 30 s, and 72 °C for 30 s; and 1 cycle of 72 °C for 60 s followed by melt curve analysis. Quantitative DENV copy number was calculated as described previously from a standard curve of Mon601 DNA. Relative RT-PCR quantitation was determined by the ∆Ct method [18] for all other genes, with PCR reactions normalized against the reference housekeeping gene glyceraldehyde-3-phosphate dehydrogenase (GAPDH).

**Table 1.** Summary of primers utilized for qRT-PCR

Name	Primer sequence	Accession no.
DENV-2	Forward: GCAGATCTCTGATGAATAACCAAC Reverse: TTGTCAGCTGTTGTACAGTCG	D00346.1
Capsid region for Mon601		
DEN-Uni-F DEN-Uni-R	Forward: *AAGGACTAGAGGTTAKAGGAGACCC* Reverse: CGYTCTGTGCCTGGAWTGATG	NC_001474.2
Universal primers for D2Y98P-PP1		
GAPDH	Forward: GACGGCCGCATCTTCTTGTGC Reverse: TGCCACTGCAAATGGCAGCC	NM_008084.3
Nos-2	Forward: GAGACAGGGAAGTCTGAAGCAC Reverse: CCAGCAGTAGTTGCTCCTCTTC	NM_001313921.1
Arg-1	Forward: CAGAAGAATGGAAGAGTCAG Reverse: CAGATATGCAGGGAGTCACC	NM_007482.3
IFN-β	Forward: AGAAAGGACGAACATTCGGAAA Reverse: CCGTCATCTCCATAGGGATCTT	NM_010510.1
Viperin	Forward: ACTCTGTCATTAATCGCTTCAACGT Reverse: TCAATTAGGAGGCACTGGAAAAC	NM_021384.4
Ifi27l2a	Forward: CTGTTTGGCTCTGCCATAGGAG Reverse: CCTAGGATGGCATTTGTTGATGTGG	NM_029803.3
IRF7	Forward: CACCCCCATCTTCGACTTCA Reverse: CCAAAACCCAGGTAGATGGTGTA	NM_001252601.1
CXCL10	Forward: GCCGTCATTTTCTGCCTCAT Reverse: GGCCCGTCATCGATATGG	NM_021274.2
CD4	Forward: CCCAGGTCTCGCTTCAGTTTG Reverse: AGGTAGGTCCCATCACCTCACAG	NM_013488.2
CD8 β	Forward: GCTGGTAGTCTGCATCCTGCTTC Reverse: TTGCTAGCAGGCTATCAGTGTTGTG	NM_009858.2

### Immunostaining and fluorescence microscopy

Cells were grown on gelatine-coated glass coverslips, infected as above, and at the indicated time point p.i. fixed in 1 % (v/v) formalin. Fixed cells were permeabilized [0.05 % (v/v) IGEPAL (Sigma)], blocked [5 % human sera, 4 % normal goat sera, 0.4 % bovine albumin sera in PBS (v/v)] and immunostained for dsRNA [J2 anti-mouse antibody (5 µg ml^−1^, 1/200 dilution), English and Scientific Consulting] and viperin [anti-rabbit antibody (5 µg ml^−1^, 1/200 dilution), Abcam]. Bound antibody was detected with donkey anti-mouse Alexa488 or goat anti-rabbit-Alexa555 and nuclei were detected with Hoechst (5 µg ml^−1^). Images were captured by florescent microscopy (Olympus, IX83).

### Tissue processing and histology

Brain tissue was fixed in 10 % (v/v) buffered formalin, embedded and block-mounted in paraffin, and 5 µm sections were cut, stained with haematoxylin and eosin (H and E) and examined by brightfield microscopy (BX53, Olympus).

### NanoString nCounter gene expression assay

Immune gene expression analysis of total RNA (WT, *n*=4 mock, *n*=4 DENV; vip^−/−^, *n*=4 mock, *n*=3 DENV) was performed using the NanoString GX nCounter Mouse Immunology Panel (NanoString Technologies, Seattle, WA, USA) on the Gen 2 nCounter FLEX Analysis System at the Systems Biology and Data Science Facility (Griffith University, Gold Coast, Australia) according to the manufacturer’s instructions. The panel contains 549 genes, including 6 positive controls, 8 negative controls and 5 housekeepers for normalization to account for platform and technical variability. Immune gene expression (nCounter) data were analysed using a combination of the Advanced Analysis Module in nSolver Analysis Software version 4.0 from NanoString Technologies (NanoString Technologies, WA, USA) and customized scripts in the R Statistical Computing Environment. Data analysis included quality control (QC), normalization and between-group comparisons for differential gene expression (DGE) and pathway analysis.

### Statistics

Data were expressed as the mean±standard deviation (sd) or ±standard error mean (sem), and statistical analyses were carried out using GraphPad Prism. A statistically significant difference between samples was determined using unpaired Student’s *t*-test, one-way or two-way analysis of variance (ANOVA) with Holm–Sidak multiple comparisons test. Kaplan–Meier survival curves were analysed by log-rank (Mantel-Cox) test. Results with *P* values of <0.05 were considered statistically significant.

## Results

### Cells lacking viperin show increased DENV replication and increased induction of type I IFN and ISG responses

**Fig. 1.**
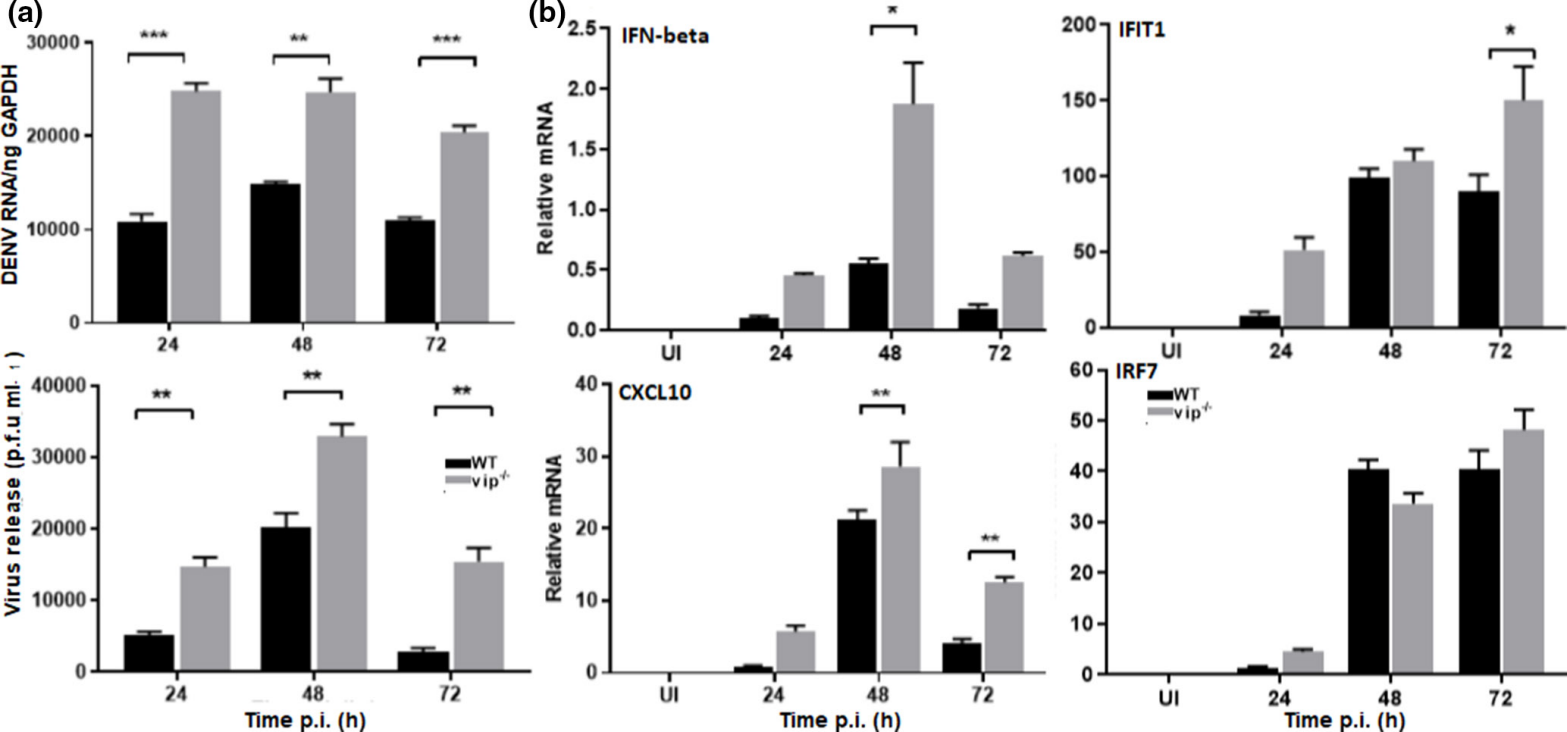
DENV infection is increased in vip^−/−^ MEFs and IFN-β and ISG mRNA are induced. MEFs were obtained from WT and vip^−/−^ mice and DENV-infected at an m.o.i. of 0.1. Samples were collected at 24, 48 and 72 h p.i. and (**a**) cellular RNA isolated and DENV copy number quantitated by RT-PCR and normalized against GAPDH (top panel); supernatant for plaque assay (lower panel); (**b**) mRNA levels for IFN-β, IFIT1, CXCL10 and IRF7 were measured by qRT-PCR and relative mRNA level quantitated by normalization against GAPDH using the ∆Ct method. Results represent mean±sem of *n*=3 assay replicates from three independent experiments. Statistical significance was determined by two-way ANOVA with Holm–Sidak multiple comparison. *P<0.05.

To assess the effect of the lack of viperin on DENV replication, MEFs were generated from WT and vip^−/−^ mice [8]. Both WT and vip^−/−^ MEFs were morphologically similar and grew at similar rates *in vitro* (Fig. S1, available in the online version of this article). WT and vip^−/−^ MEFs, both at low passage (<2 weeks culture), were DENV-infected, and productive infection was quantitated. DENV RNA levels demonstrated a significant increase in DENV-infected vip^−/−^ compared to WT MEFs across all time points p.i. (Fig. 1a). Similarly, infectious virus release was significantly increased by approximately 0.5 log in DENV-infected vip^−/−^ MEFs compared to WT cells at 24 and 48 h p.i., and nearly 1 log by 72 h p.i. (Fig. 1a). Viperin is reported to regulate the production of type I IFN in pDCs in response to viral infection [19] and thus induction of IFN-β and concomitant ISG mRNA levels were defined. Levels of mRNA for IFN-β and ISGs (IFIT1, CXCL10 and IRF7) were significantly affected by DENV infection in both WT and vip^−/−^ MEFs compared to uninfected controls (Fig. 1b). IFN-β mRNA levels tended to be higher in vip^−/−^ than WT MEFs and were significantly greater at 48 h p.i. (Fig. 1b). Similarly, mRNA for IFIT1 and CXCL10 was significantly higher in DENV-infected vip^−/−^ in comparison to WT MEFs at 72 h p.i., and 48 and 72 h p.i., respectively (Fig. 1b). Although IFN-β and other ISGs were elevated, IRF7 mRNA was induced by DENV infection but was not different in WT compared to vip^−/−^ MEFs (Fig. 1b). Collectively, these results suggest that a lack of viperin results in elevated DENV replication and that this may subsequently result in more significant induction of ISG expression.

### Mice lacking viperin are not susceptible to enhanced infection following systemic or intracranial DENV challenge

**Fig. 2.**
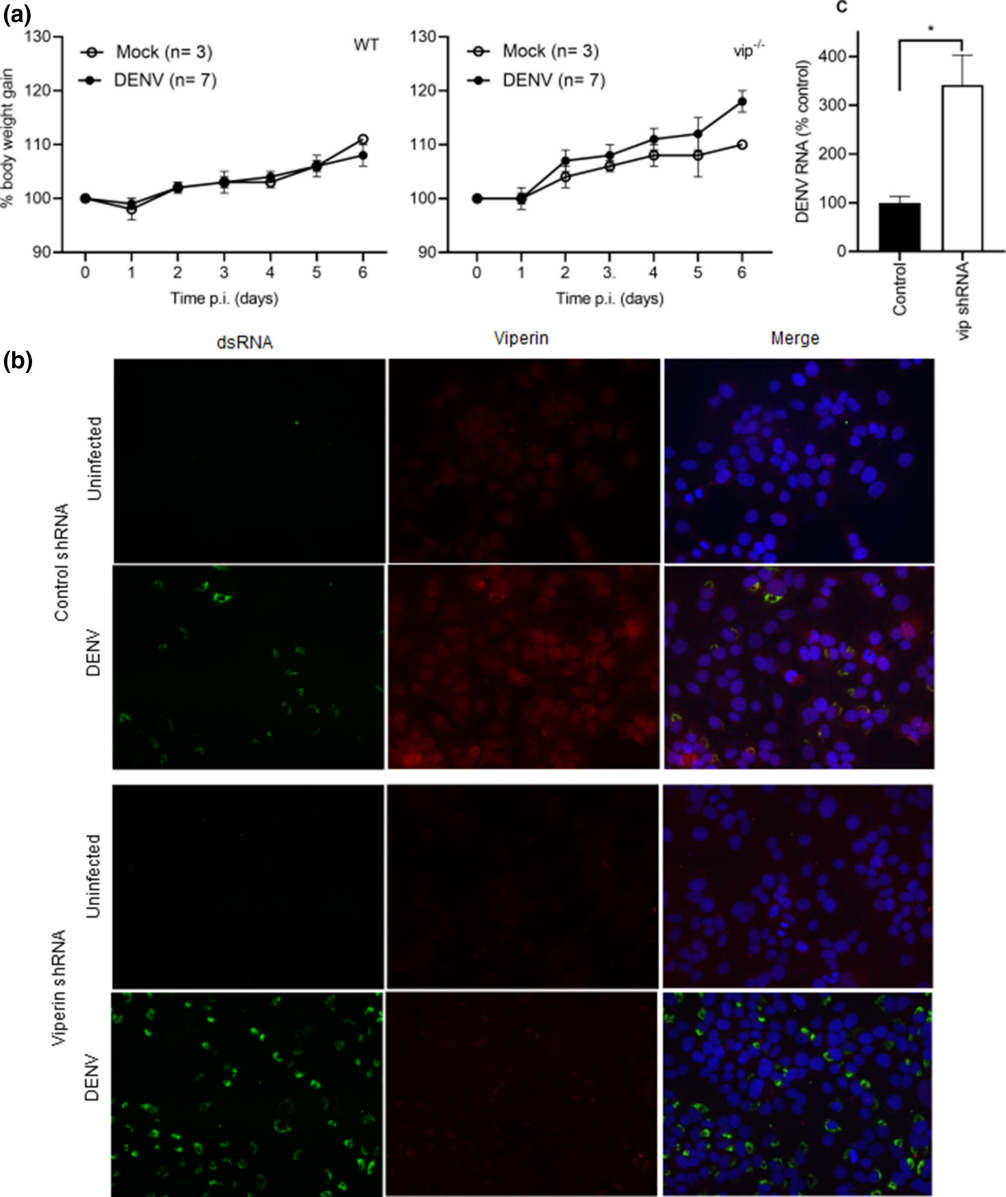
Body weight gain and survival following subcutaneous DENV infection is not affected by lack of viperin. (**a**) Five-week-old C57BL/6 WT and vip^−/−^ mice were injected subcutaneously with 10^4^ p.f.u.^−1^ DENV-2 D2Y98P-PP1. Body weight was recorded and is expressed as a percentage of starting body weight, WT (left panel) and vip^−/−^ (right panel), mock (*n*=3) and DENV (*n*=7) for both; Huh7 cells expressing a control or viperin shRNA were DENV infected at an m.o.i. of 0.5 and (**b**) at 24 h p.i. cells were fixed and co-immunostained for DENV dsRNA (green) and viperin (red) with images captured by fluorescence microscopy; (**c**) at 48 h p.i. RNA was isolated and DENV copy number quantitated by RT-PCR and normalized against GAPDH using the ∆Ct method. Results represent mean±sem of *n*=3 biological and *n*=6 assay replicates. Statistical significance was determined by Student’s *t*-test. *P<0.05.

Systemic DENV infection and disease in immunocompetent mice is restricted but DENV can infect mice lacking components of the innate immune response [20–22]. Based on this observation and our *in vitro* data, experiments assessed if similarly, a lack of viperin would increase susceptibility to systemic DENV challenge. WT and vip^−/−^ mice were subcutaneously challenged with D2Y98P-PP1, a DENV-2 strain that replicates systemically in AG129, IFN-signalling deficient mice [23]. No significant change in body weight or onset of clinical signs of infection were apparent in either WT or vip^−/−^ mice for up to 6 days p.i. (Fig. 2a), a time point at which disease is apparent in the AG129-DENV infection model [23, 24]. At 6 days p.i., samples were collected and analysed for DENV RNA by qRT-PCR. No DENV RNA or induction of viperin, as a surrogate indicator of infection, was observed in WT or vip^−/−^ blood, liver or brain (data not shown). These results suggest that loss of viperin does not result in susceptibility of mice to systemic DENV infection. To demonstrate that the mouse-adapted DENV-2 D2Y98P-PP1 strain is susceptible to anti-viral actions of viperin, Huh7 cells expressing control or viperin shRNA were infected, as in Fig. 1, and DENV infection and viperin expression were analysed (Fig. 2b). Viperin was induced in a DENV-infected Huh7 control but poorly in viperin shRNA-expressing cells. Viperin expression co-localized with dsRNA in putative replication complexes, as we have shown previously [11]. Lower viperin corresponded with a clear increase in the number of DENV-infected cells in viperin shRNA-expressing cells (Fig. 2b). Additionally, RNA was extracted and DENV RNA quantitated by RT-PCR. The results demonstrate significantly higher DENV RNA in viperin shRNA-expressing cells compared to control shRNA cells (Fig. 2c).

**Fig. 3.**
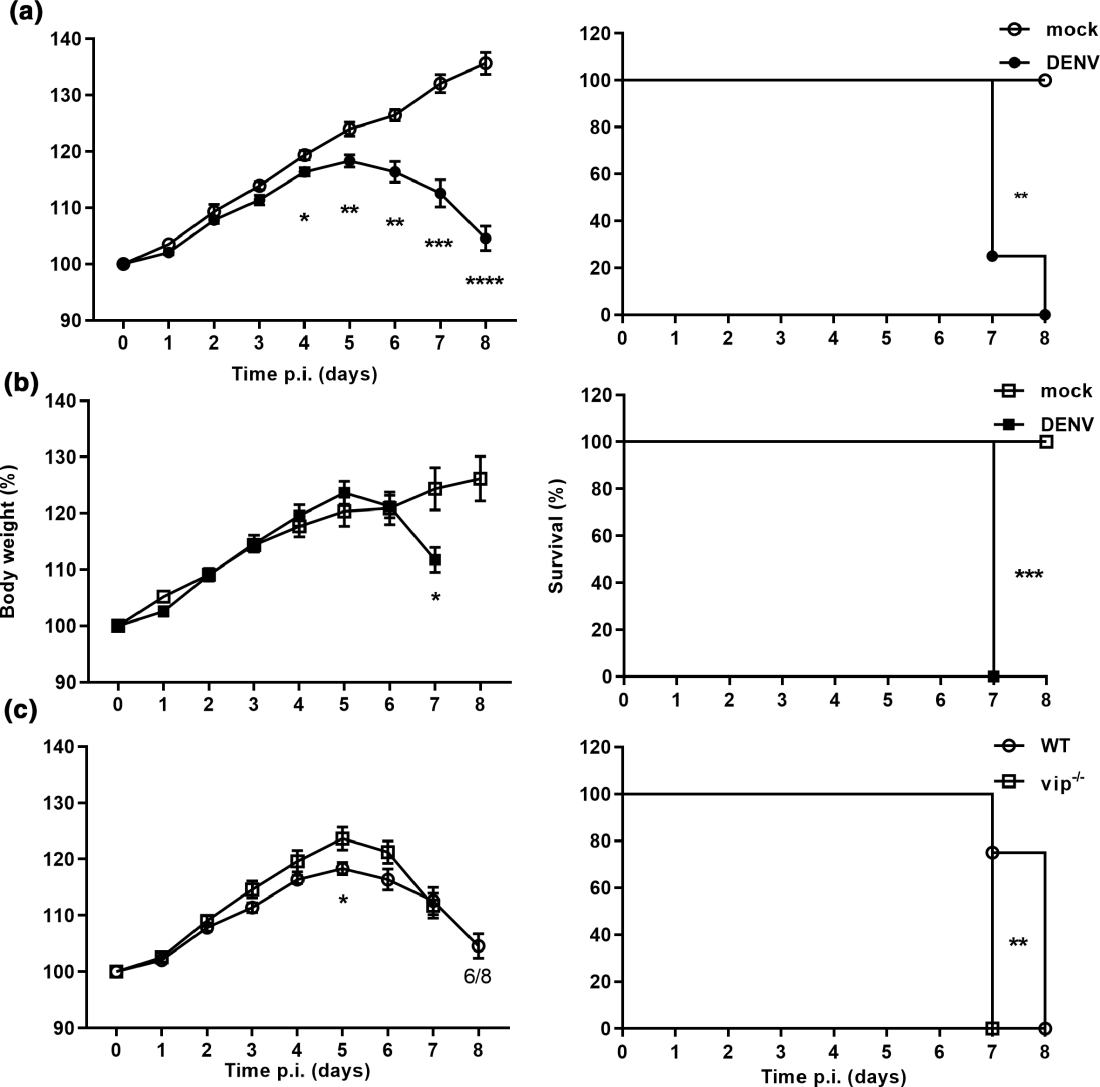
Body weight gain and survival following i.c. DENV infection is not affected by lack of viperin. Three–fourweek-old C57BL/6 WT (*n*=8) and vip^−/−^ (*n*=8) mice were i.c. injected with 800 p.f.u. ml^−1^ DENV-2 Mon601 strain. Mock WT (*n*=4) and vip^−/−^ (*n*=4) control mice were i.c. injected with vehicle only. Body weight and neurological signs were recorded. Body weight (left panel) is expressed as a percentage of initial body weight. Survival (right panel) reflects mice that do not show neurological symptoms or >10 % of body weight loss. Comparison of body weight and survival curves of (**a**) mock with DENV-infected WT mice; (**b**) mock with DENV-infected vip^−/−^ mice; (**c**) DENV-infected WT and vip^−/−^ mice. Data are expressed as mean±sem. Statistical significance for body weight was determined using two-way ANOVA and Sidak multiple comparison test. Statistical analysis of survival curves were determined by log rank test. *P<0.05.

Next, mice were subjected to direct i.c. DENV challenge, a model where replication can ensue in WT immunocompetent mice [12, 25]. WT and vip^−/−^ mice were i.c. mock- or DENV-infected and body weight gain and neurological signs were monitored. WT mice showed a significant reduction in body weight gain by 4 days p.i. (Fig. 3a, left panel) and 50 % of mice began to lose weight by 6 days p.i. (Table 2). In contrast, vip^−/−^ mice did not show a significant reduction in body weight gain until 7 days p.i. (Fig. 3b, left panel), although mice began to lose weight by 5–6 days p.i. (Table 2). As expected, the mortality rate of DENV-infected WT and vip^−/−^ mice was significantly higher compared to the respective mock-infected controls (Fig. 3a, b, right panels). Comparison of body weight gain between DENV-infected WT and vip^−/−^ mice showed no significant difference, except at 5 days p.i., where WT mice demonstrated significantly lower body weight gain than vip^−/−^ mice (Fig. 3c, left panel). At 7 days p.i., all vip^−/−^ and six out of eight WT DENV-infected mice were humanely killed due to ethical end-point of weight loss or neurological signs (Fig. 3b, c). This is reflected by a significantly higher mortality rate for DENV-infected vip^−/−^ mice at 7 days p.i. compared to WT mice (Fig. 3c, right panel). There was no significant difference in the overall number of mice that lost body weight, the time of onset of body weight loss, or the percentage of mice that lost more than 10 % of body weight (Table 2).

**Table 2.** Signs of DENV infection are not affected by lack of viperin. Summary of body weight loss and DENV-induced neurovirulence in WT (*n*=8) and vip^−/−^ (*n*=8) mice. Data are analysed using Fisher’s exact test

	Mouse strain				
	WT		vip^−/−^		*P*-value
	No.	%	No.	%	
**Body weight loss**					
Weight loss	8	100	8	100	0.999
No weight loss	0	0	0	0	
**% Body weight loss**					
≥10 %	6	75	6	75	0.999
<10 %	2	25	2	25	
**Days p.i. onset loss**					
>6 days p.i.	4	50	8	100	0.076
<6 days p.i.	4	50	0	0	
**Neurovirulence**					
Yes	3	37.5	4	50	0.999
No	5	62.5	4	50	

**Fig. 4.**
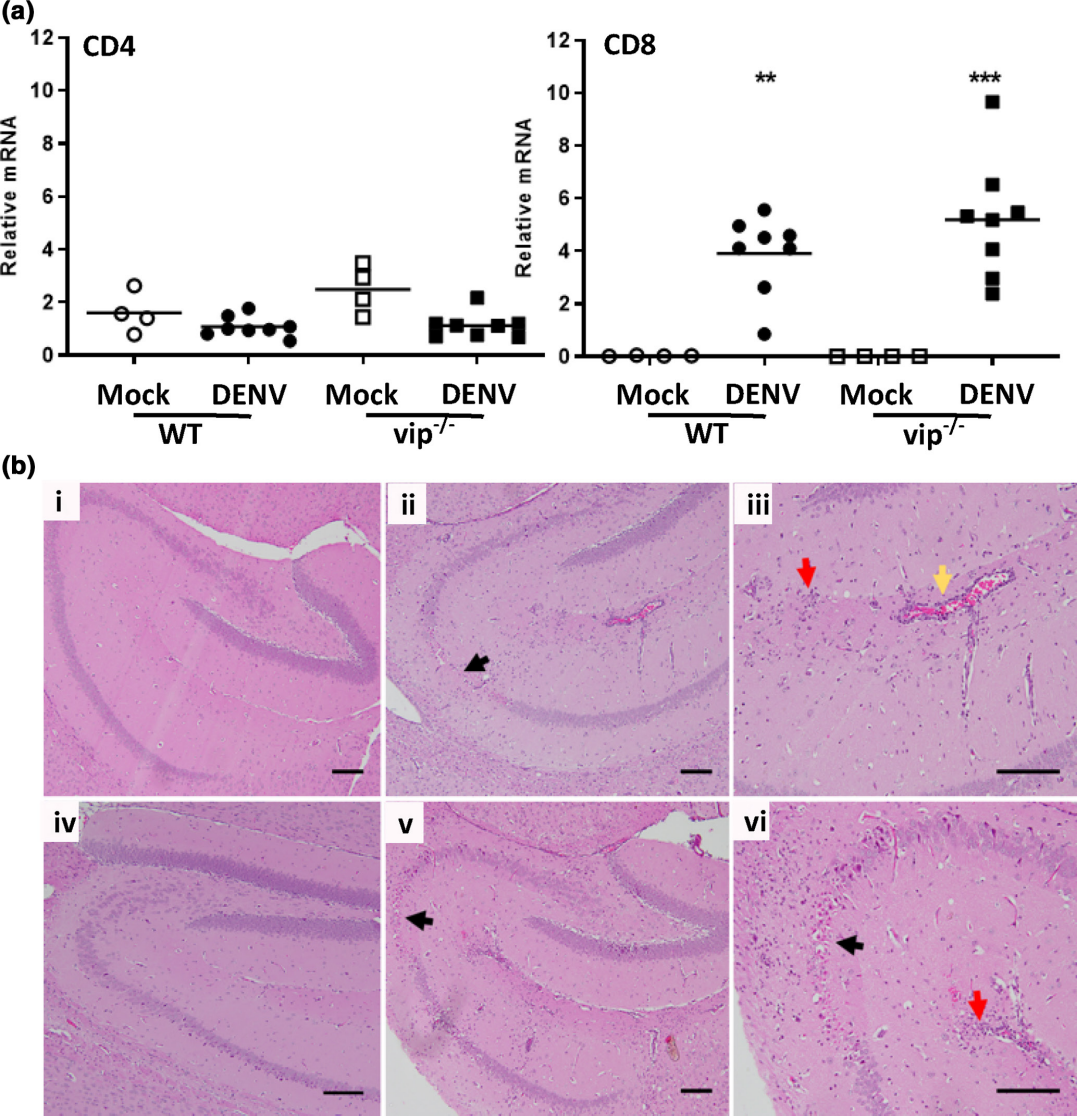
No difference in CD4^+^ or CD8^+^ infiltration or histopathological staining in the brain of WT and vip^−/−^ mice following i.c. DENV infection. WT and vip^−/−^ mice were i.c. DENV injected and at end stage disease, brains were harvested. (**a**) RNA extracted and subjected to real-time qRT-PCR for CD4 and CD8 mRNA. *n*=8 WT and vip^−/−^ DENV-infected, *n*=4 WT and vip^−/−^ mock-infected mice. Data represent average PCR values from individual mice and normalized against GAPDH by the ΔCt method. Statistical significance was assessed by unpaired two-way ANOVA with Holm–Sidak multiple comparison, *P<0.05. (**b**) Fixed and subjected to H and E staining. (**i, v**) Normal histology of mock brain hippocampus; (ii, iv) DENV-infected brain showing destruction of neurons of hippocampus region (black arrow), infiltration of immune cells (red arrow) and perivascular cuffing (yellow arrow) WT (**i and ii**) and vip^−/−^ (iv and v) with (iii and vi) higher magnification of (ii and v). Images were captured by a BX53 brightfield microscope and are representative of *n*=2 mock or DENV-infected WT and vip^−/−^ mice. Bars indicate a 100 µm scale.

DENV infection in mouse brain has been reported to induce neuroinflammatory damage in the hippocampus [26–28] and T-cell infiltration in the cortical area of the mouse brain [12]. Analysis of infiltrating CD4^+^ and CD8^+^ cells by qRT-PCR demonstrated that, consistent with previous findings [12], CD8 but not CD4 mRNA levels were significantly induced in both WT and vip^−/−^ mice following DENV infection compared to mock-infected controls (Fig. 4a). CD4 or CD8 mRNA levels were not significantly different between DENV-infected WT and vip^−/−^ mice. Histological changes in the brain demonstrated pathological changes within the hippocampus characterized by destruction of neurons, perivascular cuffing and infiltration of inflammatory cells in both DENV-infected WT and vip^−/−^ mice (Fig. 4b). Based on the qRT-PCR results in Fig. 4a, this cellular infiltrate is likely CD8^+^ T cells. There was, however, no clear difference in WT and vip^−/−^ mouse brains (Fig. 4b). This observation indicates that the lack of viperin does not alter the overall brain morphology or CD4^+^ and CD8^+^ T cell infiltration following DENV infection.

**Fig. 5.**
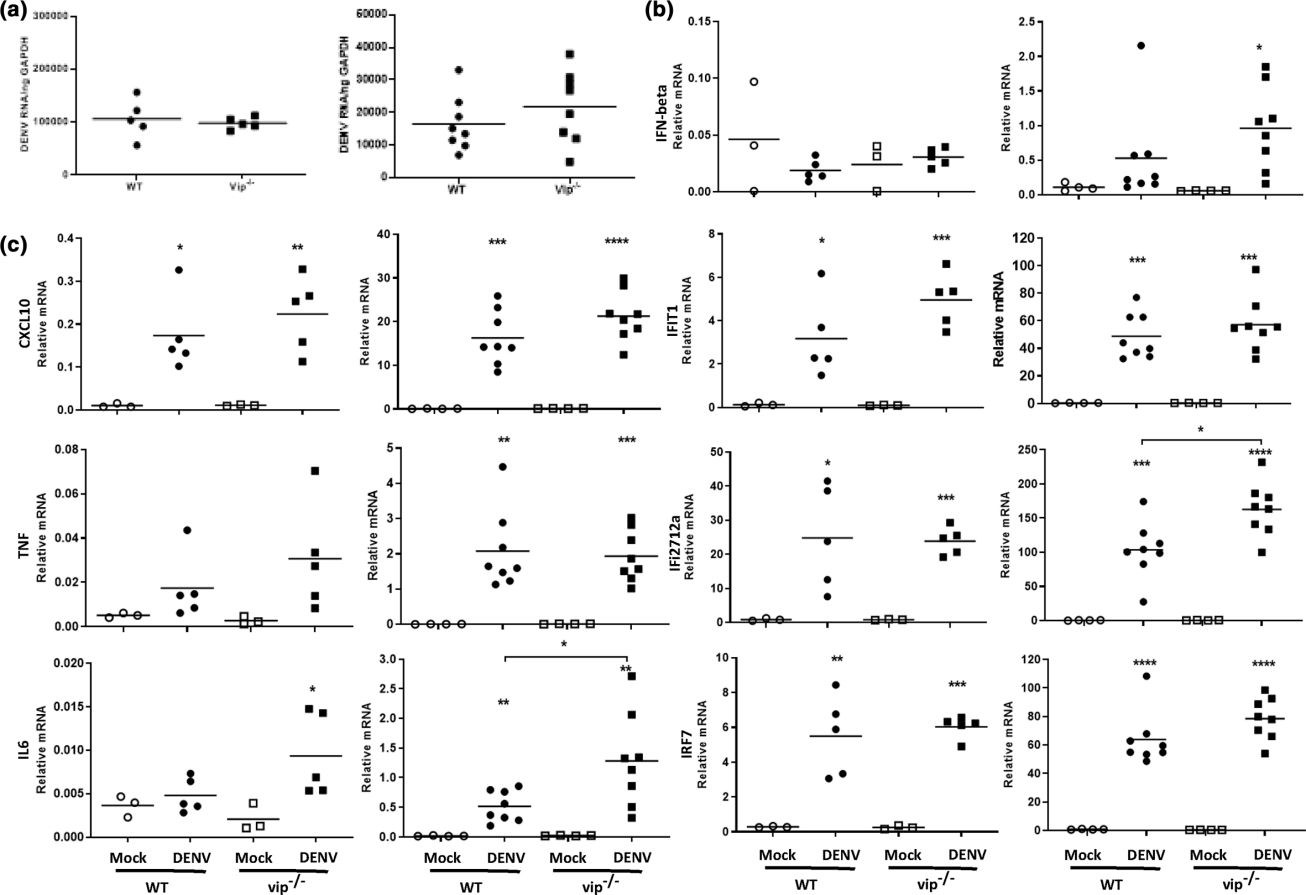
DENV RNA and ISG mRNA levels are not different in WT and vip^−/−^ mice following i.c. DENV infection. WT and vip^−/−^ mice were i.c. infected with DENV and RNA was extracted from infected brain tissue at 3 days p.i., *n*=5, for each mice strain, and at the time of humane euthanasia, representing 7 (*n*=2) and 8 (*n*=6) days p.i. for WT and 7 (*n*=8) days p.i. for vip^−/−^ mice. RNA was subjected to a real-time qRT-PCR for (**a**) DENV; (**b**) IFN-β, IFIT1, Ifi27l2a and IRF7; (**c**) CXCL10, TNF-α and IL-6, at 3 days p.i. (left panel) and end stage (right panel). Each symbol represents an individual mouse sample. Data represent average PCR values from individual mice and normalized against GAPDH by the ΔCt method. Statistical significance was assessed by unpaired two-way ANOVA with Holm–Sidak multiple comparison, *P<0.05.

The level of DENV replication in the brain of WT and vip^−/−^ mice was quantitated by qRT-PCR at 3 or 7–8 days p.i. (ethical end-stage disease). As expected, DENV RNA was not detected in the brain of mock-infected mice. No significant difference was observed in DENV RNA levels at 3 or 7–8 days p.i. between DENV-infected WT and vip^−/−^ mice (Fig. 5a, left and right panels, respectively). Further, the mRNA levels for IFN-β and ISGs were analysed by qRT-PCR, including Ifi2712a, reportedly associated with anti-viral actions against WNV in neurons in specific regions of the brain [29]. At 3 days p.i., although IFN-β is not induced, ISGs are significantly upregulated following DENV infection in both WT and vip^−/−^ mice (Fig. 5b, left panel). At ethical end-stage disease (7–8 days p.i.), IFN-β and ISGs were induced in both DENV-infected WT and vip^−/−^ mice (Fig. 5b, right panel). The levels of IFN-β, IFIT1 and IRF7 were the same in DENV-infected WT and vip^−/−^ mice, while mRNA levels of Ifi27l2a were modestly, but significantly, higher in DENV-infected vip^−/−^ compared to WT mice (Fig. 5b). Similarly, the ISG and chemokine, CXCL10 was induced early (3 days p.i.) and late in infection (7–8 days p.i.) but did not differ between WT and vip^−/−^ mice (Fig. 5c, left and right panels, respectively). mRNA levels for two proinflammatory cytokines, TNF-α and IL-6, previously shown to be increased in DENV infection [12], were analysed by qRT-PCR. At 3 days p.i. no significant induction was observed for TNF-α and IL-6 mRNAs in DENV-infected compared to mock-infected WT mice (Fig. 5c, left panel). At end-stage disease (7–8 days p.i.), mRNA levels of TNF-α and IL-6 were significantly induced in both DENV-infected WT and vip^−/−^ mice compared to mock-infected mice (Fig. 5c, right panel), with IL-6 induction demonstrating a small but significant increase in DENV-infected vip^−/−^ compared to WT mice (Fig. 5c, right panel). These data are indicative of a largely dispensable role for viperin in induction of IFN-β, ISGs and inflammatory cytokines in the brain.

**Fig. 6.**
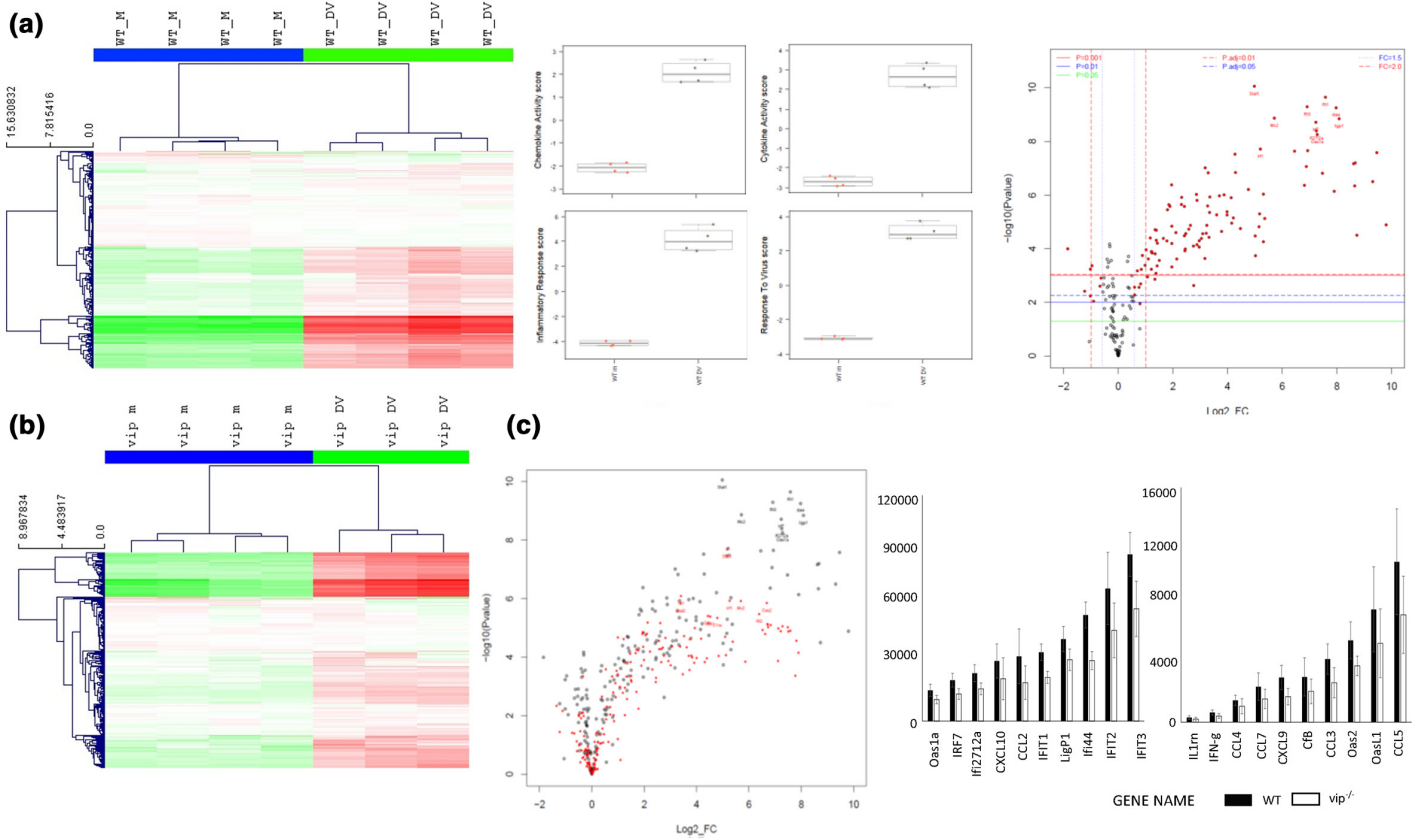
DENV infection induces inflammatory responses, but these are not different in WT and vip^−/−^ mouse brain. Total RNA was extracted from WT (*n*=4 mock; *n*=4 DENV) and vip^−/−^ (*n*=4 mock; *n*=3 DENA) mouse brain and subjected to NanoString nCounter analysis against a panel of mouse inflammatory-related genes. (**a**) Hierarchical clustering and heat map (left), pathway analysis (centre) and volcano plot (right) of results from WT samples; (**b**) hierarchical clustering and heat map analysis of results from vip^−/−^ samples; (**c**) volcano plot overlay of results from WT (grey) and vip^−/−^ (red) samples, and normalized mRNA level for the top 20 significantly upregulated genes identified in DENV-infected WT compared to DENV-infected vip^−/−^ samples. Results represent mean±sd and *y*-axes indicates raw nCounter intensity values.

LGTV demonstrates a time-dependent and early need for viperin in restricting the spread of infection in the brain [16]. To further characterize the innate responses before the onset of neurological signs but when DENV RNA levels are high [12, 25], WT and vip^−/−^ mice were i.c. mock- or DENV-infected and at 6 days p.i., RNA was harvested for NanoString nCounter analysis. The NanoString panel consisted of mouse genes associated with inflammation and full normalized data sets are available (Table S1). Unbiased non-hierarchical clustering demonstrates separate grouping of mock- and DENV-infected samples from both WT and vip^−/−^ mice and clear upregulation of mRNA for many genes in the panel (Fig. 6a, b). As expected, pathway analysis indicated upregulation of mRNA for pathways, such as chemokine activity, cytokine activity and response to virus, in DENV-infected WT (Fig. 6a) and vip^−/−^ mouse brain (data not shown). Volcano plot analysis demonstrates very few significantly downregulated mRNA, and many significantly upregulated mRNA in DENV-infected WT samples (Fig. 6a). Comparable responses were observed in vip^−/−^ samples, although the fold change in mRNA levels following DENV infection tended to be lower but not significantly different, in the vip^−/−^ samples compared with WT (Fig. 6c), and was confirmed by direct comparison of the top 20 upregulated mRNA (Fig. 6c).

**Fig. 7.**
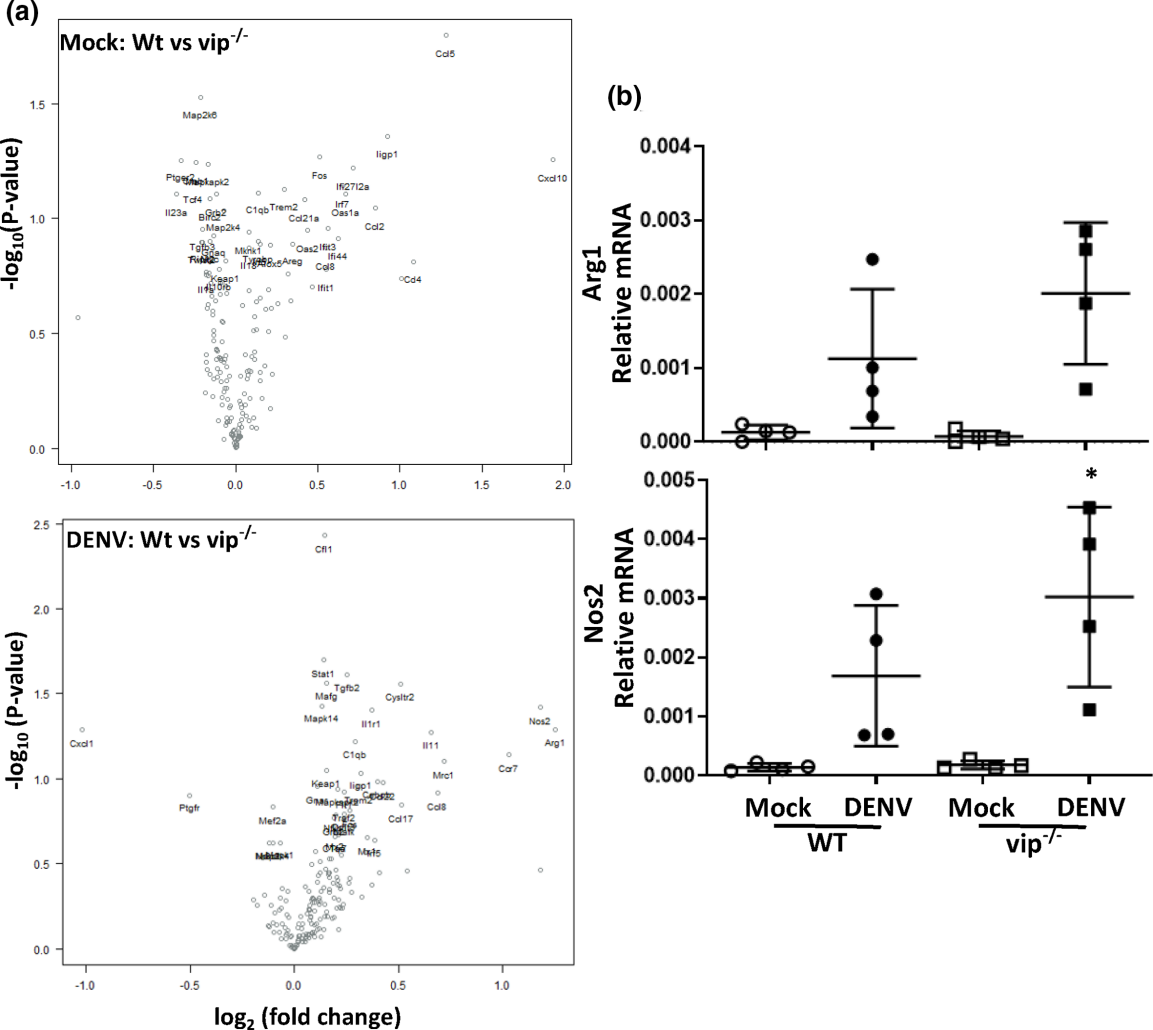
DENV infection induces Arg-1 and Nos2, but these are not different in WT and vip^−/−^ mouse brain. RNA was extracted and subjected to NanoString nCounter analysis as in Fig. 5 and responses were compared between DENV-infected WT and vip^−/−^ samples. (**a**) Volcano plot analysis for mock- (top panel) and DENV-infected (bottom panel) cases and (**b**) qRT-PCR for Arg-1 and Nos-2. Data represent average PCR values from individual mice and normalized against GAPDH by the ΔCt method. Statistical significance was assessed by unpaired two-way ANOVA with Holm–Sidak multiple comparison, *P<0.05.

No significant differences were observed in mock-infected WT or vip^−/−^ mRNA levels (Fig. 7a). Although not significantly different, Arg-1 and Nos-2 appeared close to 1.5-fold differentially expressed in DENV-infected WT and vip^−/−^ samples (Fig. 7a). This was specifically assessed by qRT-PCR, confirming the induction of Arg-1 and Nos-2 mRNA in DENV-infected mouse brain, but no significant difference in WT compared to vip^−/−^ (Fig. 7b).

**Fig. 8.**
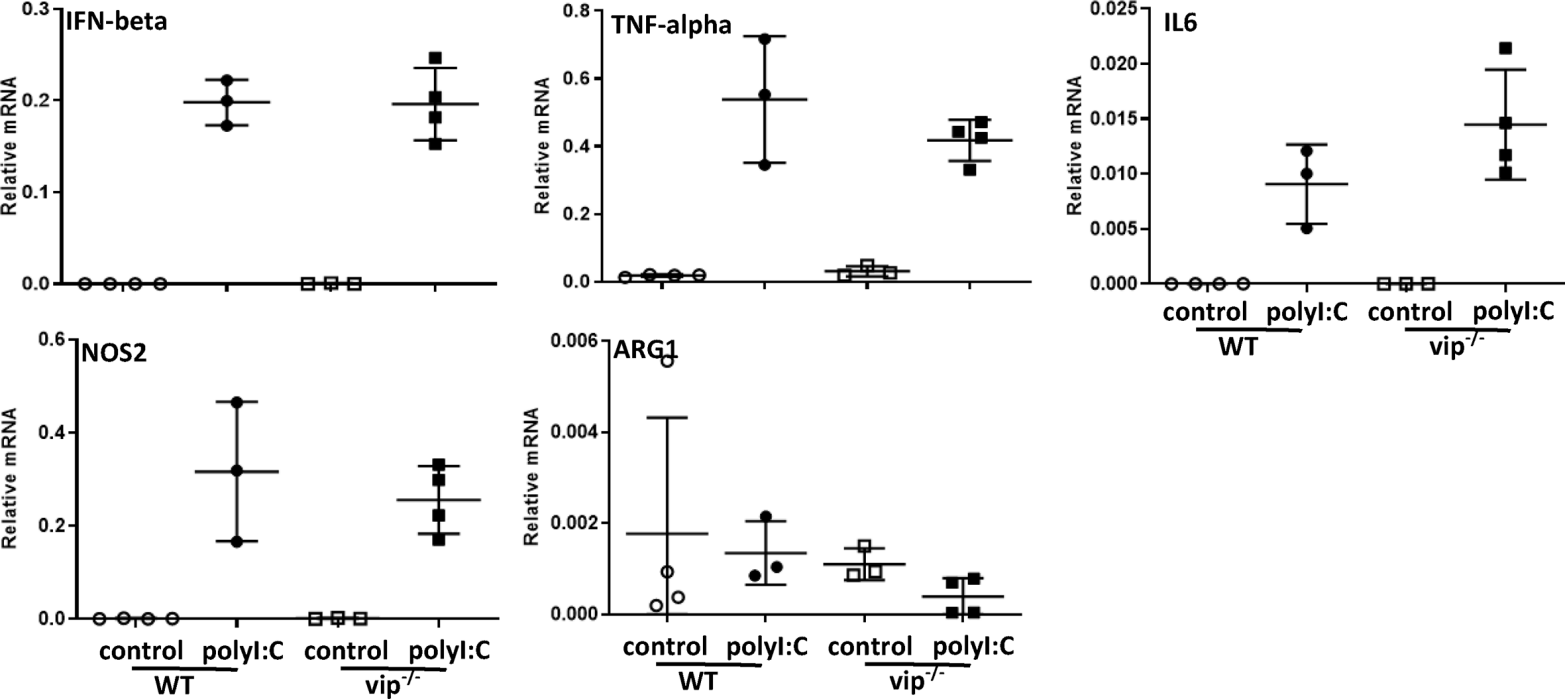
Inflammatory responses of BMDMs to the dsRNA mimic poly I:C are not different in the absence of viperin. BMDMs were generated from WT and vip^−/−^ mice and stimulated *in vitro* with poly I:C and at 24 h post-stimulation, RNA was extracted and subjected to real-time qRT-PCR for TNF-α, IFN-β, Arg1, IL6 and Nos2. Values represent mean±sem from *n*=3 (control, unstimulated) or *n*=4 (poly I:C stimulated) samples normalized against GAPDH by the ΔCt method. Statistical significance was assessed by unpaired two-way ANOVA with Holm–Sidak multiple comparison, *P<0.05.

The responses of BMDMs lacking viperin reportedly differ when stimulated with LPS/IFN-γ or IL-4, including in the induction of IL-6, Arg-1 and iNOS [30]. Thus, BMDMs were generated from WT and vip^−/−^ mice, stimulated with polyI:C to mimic an RNA virus stimulus such as DENV, and mRNA levels were quantitated. The results show the induction of TNF-α, IFN-β, IL-6 and Nos-2 following treatment with polyI:C but no significant difference in vip^−/−^ compared to WT cells (Fig. 8). mRNA for Arg-1 was not induced by polyI:C treatment in either WT or vip^−/−^ BMDMs (Fig. 8).

## Discussion

The ability of IFN to eliminate viral infection is mediated by the transcription of hundreds of ISGs such as viperin [31–33] as part of the early immune response against a range of viral infections [2, 3]. Data from our laboratory suggest a role of viperin in restricting DENV infection *in vitro* using primary human cells [11, 34], and that viperin is induced in MEFs [12, 25, 35] and in the brain and eye of mice in response to DENV infection [13]. Here, the role of viperin against DENV infection was investigated in a mouse model with a CRISPR/Cas-induced deletion in the *Rsad* gene encoding viperin [8], using MEFs *in vitro* and subcutaneous and intracranial DENV *in vivo* challenge systems in an immunocompetent background.

The results show that DENV infection is significantly increased in MEFs lacking viperin, consistent with ZIKV infection in this same viperin null mouse line [8]. Similarly, but in a different viperin null mouse model, CHIKV infection is increased in vip^−/−^ compared to WT MEFs [5]. In contrast, although vip^−/−^ BMDMs and bone marrow dendritic cells (BMDCs) are more susceptible to WNV infection, vip^−/−^ MEFs are not [14]. In human cells, siRNA-mediated knockdown of viperin enhances HIV-1 infection [36] and increases production of equine infectious anaemia virus (EIAV) in monocyte-derived macrophages (MDMs) [37]. Targeting of viperin mRNA using shRNA enhances the production of Sindbis virus (SIN) [38] and increases DENV release in Huh7 cells [4] but not JEV in human A549 carcinoma cells [38]. Similarly, MEFs lacking viperin did not show enhanced infection with TBEV or LGTV but replication was viperin-dependent in neuronal cultures from different regions of the brain [16]. Thus, outcomes for infection in the absence of viperin differ and may be influenced by both the virus and cell-type challenged.

In our study, the induction of IFN-β, IFIT1 and CXCL10 was significantly higher in vip^−/−^ compared to WT MEFs following DENV infection at 48–72 h p.i., which correlated with increased DENV RNA at 24–72 h p.i. Consistent with this, CHIKV-infected Rsad2^−/−^ MEFs showed higher production of IFN-α and β compared to WT MEFs [5]. These results suggest that in the absence of viperin, replication increases, which subsequently induces higher levels of IFN-β and potentiates the later induction of downstream ISGs. Interestingly, DENV induction of IRF7, another ISG, was not increased in vip^−/−^ compared to WT MEFs even in the face of increased IFN-β. In contrast to these *in vitro* results, DENV infection *in vivo* was not influenced by lack of viperin. Similarly, there is only a slight increase in the mortality of vip^−/−^ mice following IAV infection in lungs [15], and systemic LGTV infection in the absence of viperin did not alter mortality [16]. These findings are in contrast to the higher susceptibility of vip^−/−^ compared to WT mice to lethal WNV infection [14], and enhanced CHIKV-induced disease following footpad infection, with higher viraemia and joint inflammation [5].

For DENV, immunocompetent mice are not highly susceptible to infection [39]; however, when lacking certain components of the innate IFN response, mice become permissive to DENV replication and disease [5, 40]. Here, lack of viperin did not affect replication or disease following systemic infection at day 6 p.i., although it is possible that the viraemia may have been increased at an earlier time point. Regardless, the study next assessed DENV infection following direct injection into the brain – a model where DENV is known to replicate [12, 28]. Following i.c. infection with DENV, both WT and vip^−/−^ mice demonstrated reduced body weight gain and signs of disease, as expected [12, 25]. No major difference in body weight gain was observed between DENV-infected WT and vip^−/−^ mice, with an apparent earlier protection against body weight loss in vip^−/−^ mice. Survival analysis showed a slightly but significantly higher mortality rate of DENV-infected vip^−/−^ mice compared to their WT counterpart, with vip^−/−^ mice succumbing to disease 1 day earlier than WT mice. The overall disease profile, however, was not dramatically different. In support of this, DENV infection by direct i.c. injection caused damage in the hippocampus region of the brain with loss of neurons and infiltration of immune cells that was histologically similar in both WT and vip^−/−^ mouse brains. Similar to the lack of altered tissue pathology in the vip^−/−^ DENV brain, the absence of viperin did not significantly alter IAV-induced pulmonary damage in a mouse model of infection [15]. In contrast, enterovirus A71 (EVA-71) replication is increased in the hippocampus of BalB/c mice in the absence of viperin [41] and there is an increase in immune cell (macrophage) infiltration in the footpads of vip^−/−^ mice following CHIKV infection [5]. Although macrophages were not specifically assessed in our study, there is no change in CD4 mRNA levels in either WT or vip^−/−^ DENV-infected mouse brain, suggesting no role for viperin in CD4^+^ T cell or CD4^+^ macrophage recruitment to the DENV-infected brain. In agreement with our results, mice lacking viperin showed similar CD4^+^ and CD8^+^ T cell accumulation in the brain in response to WNV infection compared to WT mice [14]. A previous study has shown that vip^−/−^ CD4^+^ T cells are defective in Th2 cytokine production (IL-4, IL-5 and IL-13) [42], linking viperin to CD4^+^ T cell function. In our DENV i.c. infection model where CD8^+^ but not CD4^+^ cells are recruited, this role for viperin is likely of less importance.

In addition to the lack of histological change, DENV RNA level at both end-stage disease (7–8 days p.i.) and before the onset of neurovirulence signs at 3 and 6 days p.i. were not significantly different in the brain of vip^−/−^ mice compared to WT mice. This is in agreement with a study showing that the IAV titre in the lungs of vip^−/−^ mice was not significantly different at early or late stages of viral infection [15]. In contrast, WNV load was greater in the brain of vip^−/−^ mice at 8 days p.i. compared to WT following i.c. infection [14]. Similarly, CHIKV showed higher viral load in the footpad of vip^−/−^ mice at 1 days p.i. compared to WT mice [5], and LGTV was increased in the brain of infected mice lacking viperin, but only in the olfactory bulb and cerebrum [16]. Taken together, these data suggest that viperin aids the control of replication of many viruses *in vivo*, including other viruses in the brain, but in the context of DENV infection in the brain, viperin seemed to be dispensable. These *in vivo* data are inconsistent with our observation in MEFs, in which DENV RNA levels were higher in vip^−/−^ MEFs compared to WT, as well as our previously published data on the anti-viral activity of viperin against DENV in Huh7 cells, MDMs [11] and ECs [34], suggesting context-specific roles of viperin. Cell type-specific antiviral actions of viperin have been suggested previously for WNV infection in the brain [29], and LGTV or TBEV infection where neurons from different regions of the brain infected *in vitro* show different requirements for viperin [16], and distinct innate immune responses, including viperin expression profile, are seen in cortical or granule cell neurons from the brain [43]. Recently, depletion of nucleotide pools and interference with mitochondrial activity have been proposed as one mechanism of viperin’s antiviral effect [44]. Differences in nucleotide pools and mitochondrial activity, or distinct innate immune responses in viral target cells, may affect the antiviral activity of viperin seen during infection in different cell types or *in vivo* contexts.

The lack of viperin also had no effect on the expression of IFN-β, IFIT1, IRF7 and CXCL10 in mouse brain at end-stage disease, or at 3 days p.i. prior to onset of DENV-induced disease. This was reflected in both qRT-PCR, as well as the profile of mRNA for mouse genes associated with inflammation at 6days p.i., as quantitated by NanoString nCounter analysis. Clear induction of multiple inflammatory pathways was seen by NanoString nCounter analysis with comparable inflammatory gene expression profiles to those reported by microarray in JEV- and WNV-infected adult mouse brain with associated encephalitis [45]. There were, however, no significant differences in the inflammatory responses between WT and vip^−/−^ mouse brains, although the overall degree of the response seemed dampened in the absence of viperin. Consistent with these results, similar induction of IFN-β was observed in both IAV-infected WT and vip^−/−^ mice lungs [15], induction of IFN-β but not IFN-α, and other ISGs, including IRF3, IRF7 and ISG15, in WT and vip^−/−^ mice following CHIKV infection in the footpad [5], with similar circulating IFN-α/β levels in mice after WNV infection in the footpad of WT and vip^−/−^ mice [46]. Thus, in contrast to the previously suggested role for viperin in ISG induction in DCs [19], these reports and our study all suggest a dispensable role for viperin in modulating host innate IFN responses to viral infections *in vivo*.

There are also studies linking viperin to IL-6 and CXCL10. There is an inverse association of viperin with IL-6, where high fat diet-induced inflammation was accompanied by increased IL-6 in white adipose tissue in vip^−/−^ compared to WT mice [47]. Additionally, a positive correlation has been described between viperin and CXCL10 in chondrocytes [48]. Further, stimulation of BMDMs from vip^−/−^ mice towards an M1 or M2 phenotype resulted in a greater induction of IL-6, CXCL10, Arg1 and iNOS [30]. This supports our finding of a small but significant increase in IL-6 at 7–8 days p.i. in vip^−/−^ compared to WT mice by qRT-PCR. This, however, was not confirmed at 6 days p.i. by NanoString nCounter quantitation and no significant changes were seen in our study in CXCL10, Arg1 and Nos2 mRNA when analysed in WT and vip^−/−^ samples from DENV-infected brain or in BMDMs from WT and vip^−/−^ mice stimulated with the TLR3 agonist, polyI:C. Overall, although there is some support in the literature for associations of viperin with induction of inflammatory responses, our study shows no major changes in the inflammatory profile when viperin is absent and challenged with DENV in the brain, or with polyI:C in BMDMs.

In conclusion, in this study we have reported contrasting outcomes for DENV infection in the absence of viperin, dependent on the model used for experimental analysis. The *in vitro* data suggested a protective role for viperin in controlling DENV infection, since the absence of viperin enhanced DENV replication. This model also demonstrated increased IFN production and subsequent induction of some ISGs, despite the lack of viperin. The *in vivo* findings, however, showed little effect from the lack of viperin during DENV challenge in the mouse, systemically or directly in the brain. Thus, viperin clearly has the ability to offer anti-viral protection against many viruses, including DENV, but in the complex *in vivo* setting these antiviral functions are dispensable for the control of DENV and induction of other innate and inflammatory responses.

## Supplementary Data

Supplementary material 1

Supplementary material 2
