# Supplementary material for: Viperin is anti-viral in vitro but is dispensable for restricting dengue virus replication or induction of innate and inflammatory responses in vivo
Source: J Gen Virol. 2021 Oct 19;102(10):001669. doi: 10.1099/jgv.0.001669 (PMC8604189; doi:10.1099/jgv.0.001669)

Supplementary Figure 1. WT and vip$^{-/-}$ MEF are comparable. WT and vip$^{-/-}$ MEF were generated and plated at $1x10^4$ cells/well and subjected to MTT assay at 0 and 48 hr post plating. Data represents average absorbance ± SD from n=3 assay wells.

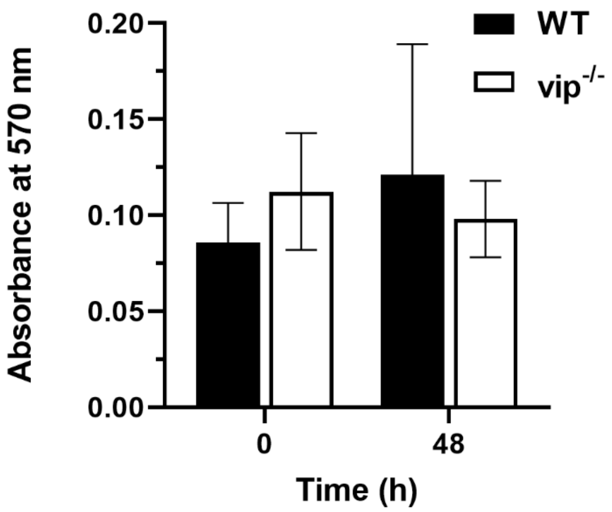

Supplement: Supplementary material 1 [file jgv-102-1669-s001.pdf]
